# Self-Healing of a Covalently Cross-Linked Polymer Electrolyte Membrane by Diels-Alder Cycloaddition and Electrolyte Embedding for Lithium Ion Batteries

**DOI:** 10.3390/polym13234155

**Published:** 2021-11-27

**Authors:** Lijuan Chen, Xisen Cai, Zhonghui Sun, Baohua Zhang, Yu Bao, Zhenbang Liu, Dongxue Han, Li Niu

**Affiliations:** 1 C/O Guangzhou Key Laboratory of Sensing Materials & Devices, Center for Advanced Analytical Science, School of Chemistry and Chemical Engineering, Guangzhou University, Guangzhou 510006, China; gdchenlj@gzhu.edu.cn (L.C.); 2111905009@e.gzhu.edu.cn (X.C.); cczhsun@gzhu.edu.cn (Z.S.); ccbhzhang@gzhu.edu.cn (B.Z.); dxhan@gzhu.edu.cn (D.H.); lniu@gzhu.edu.cn (L.N.); 2 State Key Laboratory of Electroanalytical Chemistry, Changchun Institute of Applied Chemistry, Chinese Academy of Sciences, Changchun 130022, China

**Keywords:** thermally self-healing, Diels-Alder reaction, covalently crosslinked, polymer electrolyte, lithium ion battery

## Abstract

Thermally reversible self-healing polymer (SHP) electrolyte membranes are obtained by Diels-Alder cycloaddition and electrolyte embedding. The SHP electrolytes membranes are found to display high ionic conductivity, suitable flexibility, remarkable mechanical properties and self-healing ability. The decomposition potential of the SHP electrolyte membrane is about 4.8 V (vs. Li/Li^+^) and it possesses excellent electrochemical stability, better than that of the commercial PE film which is only stable up to 4.5 V (vs. Li/Li^+^). TGA results show that the SHP electrolyte membrane is thermally stable up to 280 °C in a nitrogen atmosphere. When the SHP electrolyte membrane is used as a separator in a lithium-ion battery with an LCO-based cathode, the SHP membrane achieved excellent rate capability and stable cycling for over 100 cycles, and the specific discharge capacity could be almost fully recovered after self-healing. Furthermore, the electrolyte membrane exhibits excellent electrochemical performance, suggesting its potential for application in lithium-ion batteries as separator material.

## 1. Introduction

In biological systems, Nature offers an amazing ability: self-healing, which can be used to confront with the dilemma of mechanical fractures [1]. Almost in all biological tissues, such as human skin, have the ability to self-repair themselves when damaged. After injuries heal, the skin is able to restore the same functions as before. The ability of self-healing can enhance significantly the lifetime of biomaterials after being damaged [2,3,4]. Inspired by Nature, the demand for self-healing materials is rapidly developing, which can offer a new strategy toward safer, longer-lasting products and lower production costs [5,6]. Nowadays, synthetic self-healing materials are able to repair themselves and recover functionalities after being subjected to a variety of injuries [7,8,9], which has been demonstrated to have encouraging prospect for applications in functional surfaces [10,11] electrical conductors [12,13,14], flexible sensing [15,16,17], and electronic skins [18,19,20], with enhanced lifetime and durability.

The self-healing ability is particularly desirable for energy storage because the lifetime of many rechargeable batteries is limited by the similar dilemma of mechanical fracturing during the cycling process [21]. Lithium ion batteries (LIBs), an important class of energy storage devices, are drawing much attention due to their high energy density, fast charge and discharge rates, and long cycle lifetimes [22]. Most of the current research on lithium ion batteries is focused on improving the safety of lithium ion batteries by constructing unique electrode materials [23,24,25], modifying electrolyte membranes [26,27,28,29], and improving electrolytes for greater compatibility with various device architectures [30,31]. However, when most of these lithium ion batteries are subjected to practical applications, the membrane materials become susceptible to structural defects in the electrode materials and electrolytes during the charge and discharge processes that then could lead to fracture of the membrane, which might trigger the explosion of the battery [32]. Thus, the properties of the membranes of lithium ion batteries play a crucial role in their safety performance [33,34]. Nowadays, lithium-ion polymer batteries are relatively satisfactory for portable electronics in terms of energy and power densities, cyclability, and safety, however, polymer electrolyte membranes can experience structural defects or mechanical damage caused by deformation during cycling or after accidental cutting. These failures would seriously limit the reliability and lifetime of the lithium ion batteries, resulting in the wholesale breakdown of the electronic devices, generation of abundant electronic waste, inconvenience, as well as safety hazards [35]. Recently, recycling the waste polymers from lithium-ion batteries has attracted wide attention. Stephan et al. have cleaned the used separators from spent LIBs with deionized water and reused them for the fabrication of new batteries. Although the used separators possessed the necessary electrochemical activity in the battery applications, but the mechanical properties, the discharge capacity and Coulombic efficiency cycle numbers were reduced by half compared to fresh separators. The possibility of reusing the spent battery separator is still a huge challenge. Hence, the development of self-healing materials in lithium-ion batteries will have great application potential in the development of next-generation high-performance lithium metal batteries [36].

At present, research on the self-healing ability of energy storage devices is focused on enabling the spontaneous repair of mechanical damage or preventing the structural fracture of the electrode materials and thus increasing the lifetime of the devices [37,38], but the self-healing ability requires additional materials, which cannot be used alone as either the electrode material or the membrane material. To some extent, these methods may also lead extra consumption. Thus, an ideal energy storage device should not only retain high capacitance and high rate, but also be endowed with intrinsic self-healing capability to repair any structural fractures or mechanical damage, and restore the devices’ configuration integrity and electrical properties. Liu et al. reviewed the development of thermally induced self-healing polymers based on Diels–Alder chemistry and other approaches such as hydrogen bonding, π–π stacking and disulfide interchange reactions [39]. Xue et al. introduced self-healing single-ion conducting polymer electrolytes (SIPEs) made by RAFT copolymerization to reduce concentration polarization, leading to the suppression of the formation of lithium dendrites and healing of cracks. The optimal proportion of PEs (SIPE-5) showed good thermal stability, comparatively high ionic conductivity and high lithium-ion transference properties [40]. Guo et al. designed and synthesized self-healable solid polymeric electrolytes (SHSPEs) with excellent electrochemical characteristics and fast self-repairing capability through a cross-linked network with dynamic intermolecular/intramolecular hydrogen bonding between PEG and PU [41]. However, the weaker mechanical properties and self-healing property, especially the healing time, should be further improved.

Therefore, an optimal method of processing a self-healing membrane would encompass a cheap, simple process, one that can be used as the polymer electrolyte membrane, and membrane materials that are endowed with self-healing properties. In this work, we report a new method for developing durable polymer electrolyte membranes with high ionic conductivity, high mechanical strength and self-healing properties. These self-healing polymer (SHP) electrolyte membranes take advantage of the high ionic conductivity of polymethylmethacrylate (PMMA), the flexibility of polybutylacrylate (PBA) and the self-healing ability of the Diels-Alder reaction groups [42]. The prepared self-healing polymer electrolyte membranes (SHPEM) exhibited self-healing performance, and the electrochemical performance of the cells with repaired electrolyte membranes are consistent with that of the original membranes. Such characteristics will not only remarkably prolong the lifetime of future energy storage devices, but also endow them with desirable economic and human safety attributes.

## 2. Materials and Methods

### 2.1. Materials

Methyl methacrylate (AR, 99%), lithium cobalt oxide (99.0% metals basis), butyl methacrylate (99%, containing 10 ppm MEHQ stabilizer), 4,4’-bismaleimidodiphenylmethane (>96%), lithium bis(oxalato)borate (≥99.0% metals basis), methacryloyl chloride (95%, containing 200 ppm MEHQ stabilizer), anhydrous tetrahydrofuran, furfuryl alcohol (AR, 98%), triethylamine and ammonium persulfate were purchased from Aladdin (Shanghai, China) and used as received.

### 2.2. Preparation of the Functional Monomer Furfuryl Methacrylate

Methacryloyl chloride (3.87 mL, 0.040 mol) was dissolved in 40 mL of anhydrous tetrahydrofuran and added dropwise to a stirred solution of furfuryl alcohol (3.53 mL, 0.040 mol) and triethylamine (5.55 mL, 0.040 mol) in anhydrous tetrahydrofuran (60 mL) at 0 °C. After complete addition, the mixture was stirred for 3 h at room temperature. The triethylamine hydrochloride precipitate was filtered off and the filtrate was concentrated under vacuum. The product was purified by silica gel flash chromatography (petroleum ether/ethyl acetate, 40:1). After the removal of the solvents, the product F-MA was isolated as a light yellow oil (yield, 81%).

### 2.3. Preparation of the Self-Healing Polymer Precursor

The P(MMA-BA-(F-MA)) copolymer was prepared by emulsion polymerization. In brief, 2.5 wt% sodium dodecyl sulfate solution was prepared (as an emulsifier) with deionized water under N_2_ at 60 °C. The mixture (MMA:BMA:F-MA = 1:2:1 (in molar ratio)) was added to the above solution under vigorous stirring for 1 h to form an emulsified solution. After adding ammonium persulfate (as an initiator) and stirring continuously for 6 h, the resulting emulsion was poured into 3 wt% Al_2_(SO_4_)_3_ solution to yield a precipitate that was isolated by filtration and washed with hot deionized water (55 °C) in order to remove any impurities such as residual monomers and emulsifier. The P(MMA-BA-(F-MA)) copolymer was finally obtained by drying the purified precipitate in a vacuum oven at 60 °C for 12 h and kept in a desiccator for membrane preparation.

### 2.4. Preparation of the Self-Healing Polymer Electrolyte Membrane

An optimal P(MMA-BA-(F-MA)):BMI-LiBOB self-healing polymer electrolyte composition was selected (the optimal molar ration of ((MMA-BA-(F-MA)):BMI = 2:1.1). Initially, a homogeneous solution of copolymer and lithium salt was prepared by mixing the components at 50 °C for 1 h. Subsequently, crosslinking agent was added to the mixture, after complete dissolution, the resulting viscous solution was cast with a doctor blade onto a glass plate, and then dried in vacuum at 90 °C for 24 h; finally, self-healing polymer electrolyte membrane was obtained.

### 2.5. Fabrication of the Self-Healing Polymer Lithium Ion Battery

Coin cells were assembled using lithium cobalt oxides (LCO) positive electrode and lithium metal negative electrode. The cathode was prepared by hand mixing LCO, acetylene black and PVDF in an 8:1:1 weight ratio. The PE membranes cells were fabricated with commercial liquid electrolyte (1 M LiPF_6_ in dimethyl carbonate (DMC)/diethyl carbonate (DEC)/ethylene carbonate (EC) (1:1:1, *v*/*v*/*v*)). The self-healing polymer electrolyte membranes were fabricated between the two electrodes and a small amount of solution (EC:DMC = 1:1, *v*/*v*, 10 μL) as the plasticizer add into the self-healing polymer electrolyte membranes. The assembled coin cells were employed to evaluate the electrochemical performance.

### 2.6. Characterizations and Electrochemical Tests

The morphologies were investigated by the scanning electron microscope (Phenom Pro, 5 kV, Phenom Scientific, Eindhoven, Noord-Brabant, Netherlands). Tensile-stress measurements of as-prepared self-healing polymer compostites were carried out by an 410R250 machine (TestResources, Shakopee, MN, USA). The thermal stability of the copolymer was determined by thermo gravimetric analysis (TGA, TG209F1) under N_2_ atmosphere from room temperature to 400 °C at a heating rate of 10 °C·min^−1^ (NETZSCH Group, Selb, Germany). Charge-discharge measurements of the coin cells were carried out on a battery test system (BTS, Neware Technology, Shenzhen, Guangdong, China) within the voltage range from 3.0 to 4.3 V. Electrochemical impedance spectroscopy (EIS) and interface stability were performed using a Solartron 1255 B Frequency Response Analyzer (Solartron Analytical Inc., Farnborough, Hampshire, UK) over the frequency range of 10^−1^ to 10^5^ Hz with 10 mV amplitude from the cell SS/SHPEM/SS and Li/SHPEM/Li with liquid electrolyte. Linear sweep voltammograms of self-healing polymer electrolyte composition obtained from the cell SS/SHPE/Li. For these electrochemical tests, the thickness of SHP electrolyte membranes and PE films are 12 μm and 15 μm, respectively. The diameter of SHP electrolyte membranes and PE films are both 16 mm.

## 3. Results

### 3.1. Synthesis and Characterization of SHP

**Figure 1:**
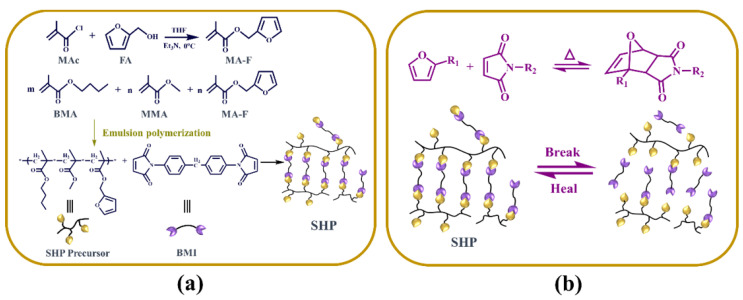
(**a**) Molecular structure design and synthesis of MMA-F, self-healing polymer (SHP) precursors and the SHP composite. (**b**) The mechanisms of the SHP composite based on the thermally reversible Diels-Alder (DA) reaction.

The monomer structures and the preparation methods of the SHP composite are illustrated in Figure 1a. By modifying methylmethacrylate (MMA) with furfuryl alcohol (FA), we can create the monomer furfuryl methacrylate (F-MA) that is not only a cross-linking group that can promote the strength of the SHP, but also a self-healing group by triggering the Diels-Alder reaction. Next, the robust flexible self-healing precursor polymer was prepared by emulsion polymerization using MMA, butyl methacrylate (BMA) and F-MA monomers with the molar ratio of 1:2:1. Then, by adding *N*,*N*’-4,4′-diphenylmethanebismaleimide (BMI) to the *N*-methylpyrrolidone (NMP) solution containing the precursor polymer and lithium bis(oxalato)borate (LiBOB), we created a crosslinked mechanically robust polymer matrix in situ to provide lithium-ion transport pathways and thermal response self-healing ability after filming and drying. Furthermore, the reversible D-A reaction was mainly affected by the temperature even in the presence of lithium salt. Figure 1b shows the self-healing mechanism of the SHP composites. The thermally reversible Diels-Alder (D-A) cycloaddition of multi-furan and maleimide groups were used to prepare the SHP composite. A highly cross-linked network can be formed via the D-A reaction of these furan and maleimide moieties, and thermal reversibility can be accomplished by the reversible D-A reaction. When are membranes damaged leading to a short-circuit, the battery would be quickly heated due to the large current flow. Thus, the fractures could be repaired by the generated heat, enabling the restoration function for the SHP composite. In principle, this process does not require additional ingredients such as catalyst, additional monomer, or special treatment of the fractured interface and could restore the fractured part of the polymer electrolyte multiple times. As a result, the as-assembled lithium ion battery should be an ideal self-healing device.

**Figure 2:**
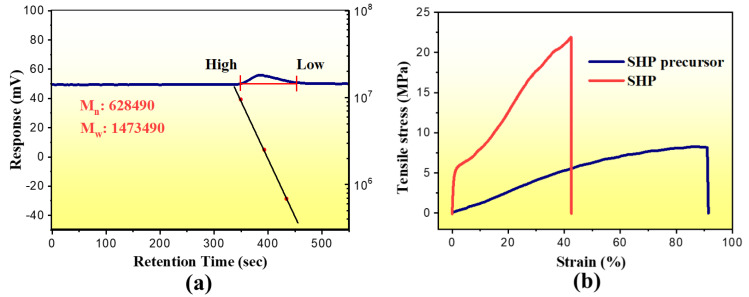
(**a**) GPC curve for self-healing polymer precursor in DMF solution. (**b**) Typical stress-strain (stress-stretch ratio) curves of SHP precursor and SHP.

Firstly, we characterized the prepared self-healing polymer composite. By an emulsion polymerization method, we have been prepared self-healing polymer precursors. Gel permeation chromatography (GPC) data shows that the number-average molecular weight of the self-healing polymer precursors reaches 644,000 (Figure 2a). We can expect that the random copolymer will have excellent physical properties due to its high molecular weight. The tensile tests show that the tensile strength of the self-healing polymer precursors reaches 5 MPa. Not surprisingly, the tensile strength of the cross-linked polymer network reached 21 MPa after adding a suitable amount of the cross-linked agent BMI as shown in Figure 2b.

**Figure 3:**
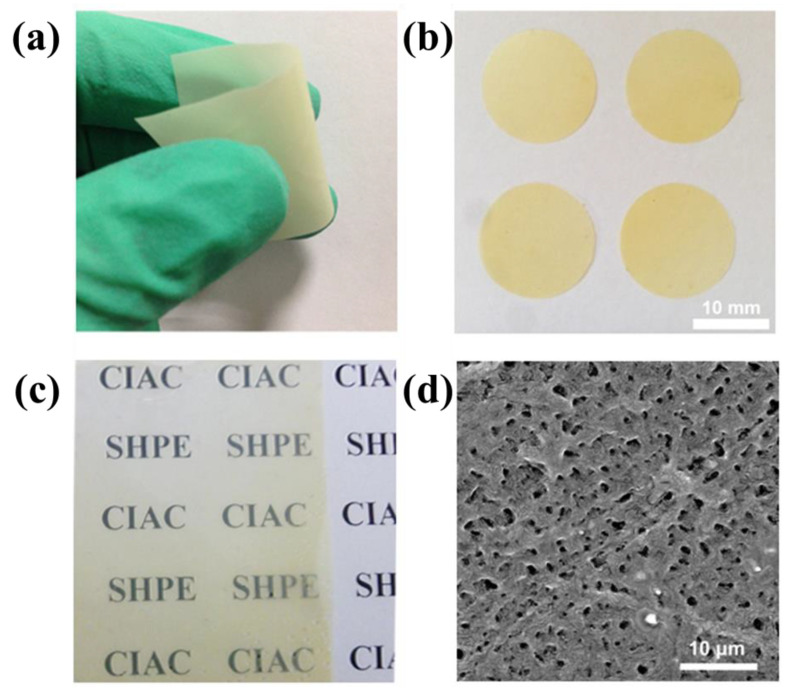
(**a**) Optical image of the flexible SHP electrolyte membrane. (**b**) Optical image of the SHP electrolyte membranes 20 μm in thickness. (**c**) The transparency of the SHP electrolyte membranes (the thickness of the membranes is 10 μm). (**d**) SEM image of the SHP electrolyte membrane.

The morphology robustness of the cross-linked polymer electrolyte is shown in Figure 3a, where its remarkable flexibility is also demonstrated. It is impressive to note that the sample is easily curved and highly flexible at a thickness of about 20 μm. Figure 3b presents an image of a prepared SHP electrolyte membrane (~20 μm thick) with 10% mass solid electrolyte loading. Phase aggregation and lithium precipitation was not observedwhen amount of added LiBOB to 10 wt%, which ensures the Li^+^ transport. The transparency of the membrane (~10 μm thick) can also be demonstrated (as shown in Figure 3c) by placing it onto the ‘CIAC SHPE’ logo. Figure S1 presents the thermogravimetric analysis (TGA) curves for the cross-linked self-healing polymer, confirming its high-temperature stability at 280 °C. Scanning electron microscopy (SEM) analysis was conducted to characterize the morphology of the cross-linked polymer films. A representative top view is shown in Figure 3d, where the surface of the cross-linked polymer presents a uniform porous structure, resulting from the fabrication method adopted. We can see that the large number of pores with an average diameter of 1.0 μm on the surface and pores are interconnected under the surface, which are necessary for the membrane to have high ionic conductivity.

### 3.2. Self-Healing and Mechanical Property of the SHP Electrolyte Membranes

**Figure 4:**
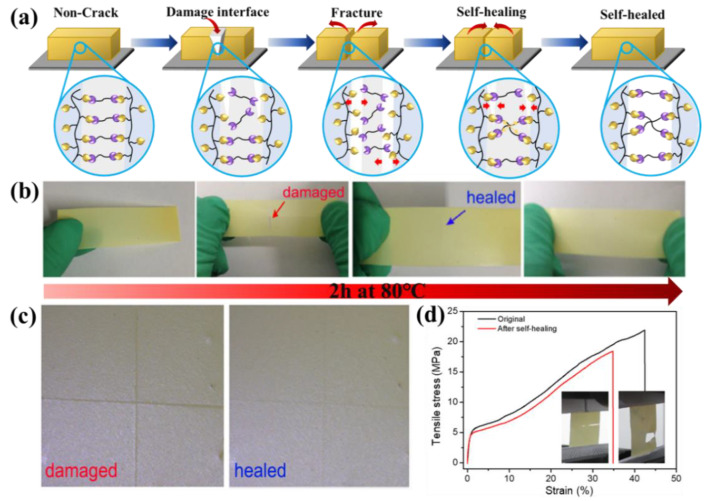
The mechanical self-healing properties of the SHP electrolyte membrane (after 2 h under 80 °C). (**a**) Schematic illustration of the self-healing process with DA reaction. (**b**) Demonstration of the healing process for the SHP electrolyte membranes (the thickness of the membranes is about 25 μm). (**c**) Optical microscopic images of a damaged sample (left) and fracture healing (right) for the damaged sample. (**d**) Tensile measurements of the SHP electrolyte membrane, and the healed sample (inset: snapshots of SHP membranes after tensile measurements).

Furthermore, the self-healing performance and mechanical property of the SHP electrolyte membranes is demonstrated in Figure 4. The SHP electrolyte membranes with furan and maleimide moieties possess excellent self-healing properties via the reversible D-A reaction at 80 °C under nitrogen for about 2 h (Figure 4a,b). Furthermore, to determine the fracture-mending efficiency of this polymer electrolyte membranes, tests were performed with the use of scars recovery and tension test specimens. At first, a cross scar was gently sliced in the samples using a fresh razor blade. After structural failure, the two pieces were matched as closely as possible and treated at 80 °C under nitrogen for about 2 h, then cooled down to room temperature. Photographs of the mending effect of a typical specimen are shown in Figure 4c. Before mending, the interface of the crack was very obvious, while after thermal treatment, the scar of the specimen was almost not observed, indicating healing of the scar. Fracture healing tests also carried out to evaluate the healing efficiency. After treatment at 80 °C under nitrogen for about 2 h, the mending efficiencies are impressive. Our results indicated that any cracks in the fracture specimen are also difficult to observe (Figure 4b). Finally, fracture tensile tests were carried out in an effort to quantify the healing efficiency. We cut the specimen into two separate pieces and repaired them into one piece. Before healing, the two pieces were matched as closely as possible and clamped. After treatment at 80 °C under nitrogen for about 2 h, the typical stress-strain curves for the original and self-healed samples are plotted in Figure 4d, showing a recovery of about 65% of the original breaking strength. This repair strength is probably due to the fact that the healed region has different mechanical properties from the original material and is a result of the unique interfacial bonding [38] because the bond strength between diene and dienophile of the D-A adduct is much lower than all the other interactions, such as C-C bonds, C-O bonds, multiple hydrogen bonds and chain entanglement interactions, and in the crack region, the reversible D-A reaction should be the major interaction. In principle, when the damaged sample is heated, the furan and maleimide moieties should reconnect, some chains should entangle again and the cracks or fractures should be mended.

### 3.3. Electrochemical Performances of SHP Electrolyte Membranes

**Figure 5:**
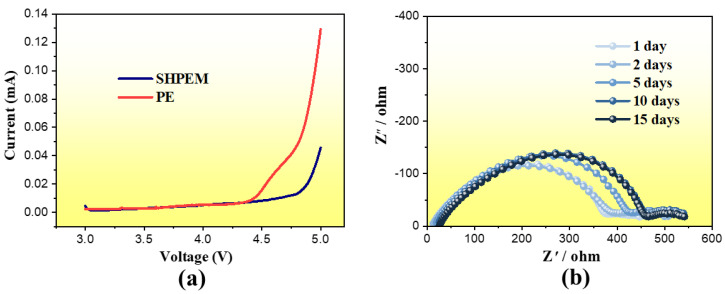
(**a**) Linear-voltammograms on stainless steel for SHPEM and commercial PE at scan rate of 5 mV·s^−1^. (**b**) Impedance spectra of cell Li/SHPEM/Li at open circuit potential.

As one of the most important experiments, the electrochemical performance of the SHP electrolyte membranes and the lithium ion battery based on the as-prepared SHP electrolyte membranes were investigated. All the SHP electrolyte membranes used in electrochemical performance tests have a thickness of about 15 μm. The electrochemical stability of the SHP electrolyte membranes was tested using the asymmetric cell stainless steel (SS)/SHPEM/Li. Linear sweep voltammograms displayed that the SHP electrolyte membranes decompose at about 4.8 V (vs. Li/Li^+^). However, the commercial PE film is only stable up to 4.5 V (vs. Li/Li^+^), as seen from the curve in Figure 5a. This indicates that the SHP electrolyte membranes has better electrochemical stability than commercial PE film. Interfacial stability of the electrode is also an essential factor to guarantee acceptable performance in the lithium ion batteries. To understand the stability of the interface between Li and SHPEM, a cell Li/SHPEM/Li was set up and AC impedance spectroscopy was used to monitor the change in impedance with time. It can be seen from Figure 5b that the resistance of the passive film increases within two weeks but remains almost unchanged after two weeks. This suggested that it takes two weeks for the formation and stabilization of the passive film on lithium. After that, the resistance of the SHPEM was hardly related to the time, the lithium does not change the performance of the SHPEM, indicating that the SHPEM have a good compatibility with lithium.

**Figure 6:**
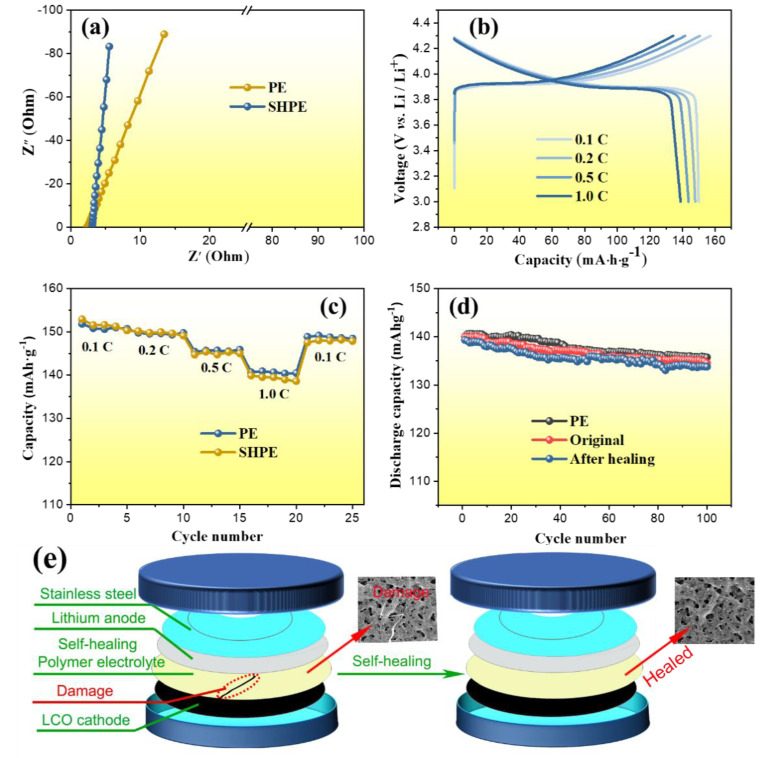
Electrochemical characterizations of the self-healing lithium ion battery. (**a**) Nyquist plots of the SS/SHPEM/SS and SS/PE/SS cells at room temperture. (**b**) Galvanostatic charge-discharge curve tests of PE membrane batteries and SHPE membrane batteries at various C-rates. (**c**) Discharge capacity of a PE membrane and a SHPE membrane cycled at various C-rates. (**d**) Cycling performance of the original SHPE membrane, healed SHPE membrane and PE membrane cycled at 1 C rate. All cells were charged to 4.3 V and discharged to 3.0 V. (**e**) The schematic of the damaged and healed SHPE membrane of lithium battery.

In order to determine the ionic conductivity, SHPEM was sandwiched between two parallel SS discs. The ionic conductivity (σ) was calculated from the bulk electrolyte resistance (R) according to the following equation: σ = l/(RA), where R is the resistance of the bulk electrolyte, l is the thickness of the film and A is the area of electrode covered by film. Figure 6a presents the plots of the ionic conductivities of SHP electrolyte membranes and PE films, and the Arrhenius plot for ionic conductivity as a function of temperature of the SHP electrolyte membranes has shown in Figure S2 (In details, the ionic conductivities of SHPEM at different temperature of 25 °C, 35 °C, 45 °C, 55 °C, 65 °C, 70 °C, 85 °C and 90 °C are 1.69 × 10^−4^, 2.69 × 10^−4^, 4.17 × 10^−4^, 6.61 × 10^−4^, 9.33 × 10^−4^, 1.32 × 10^−3^, 1.86 × 10^−3^ and 2.34 × 10^−3^ S·cm^−1^, respectively). Furthermore, it can be seen from Figure 6a that the imaginary part of the impedance is linearly related to its real part. The intersection of the straight line with the real part axis is the bulk electrolyte resistance (R). The ionic conductivity of the SHP electrolyte membranes with small amount of liquid electrolyte (10 μL) is approximate to 2.56 × 10^−3^ S·cm^−1^ at room temperature, which is a little lower than that of the commercial PE films (2.76 × 10^−3^ S·cm^−1^). The high ionic conductivity of the SHP electrolyte membrane should be ascribed to the interconnected pores in SHP electrolyte membrane, the high content of LiBOB and the microstructure of the amorphous phase.

Finally, the SHPEM was investigated as a functional separator in a lithium-ion battery. Figure 6b shows the charge-discharge curves of the battery Li/SHPEM/LiCoO_2_ at different current rates (0.1 C to 1 C). On galvanostatic cycling between 4.3 V and 3.0 V, the charge capacity reached 158 mAh·g^−1^ for the first cycle and the discharge capacity reached 149 mAh·g^−1^ at a constant current of 14 mA·g^−1^ (0.1C rate), delivering a first-cycle efficiency of 94.30%, indicating an excellent energy efficiency. The specific capacities of the Li/SHPEM/LiCoO_2_ batteries achieved 139 mAh·g^−1^ under 1 C rate, so the capacity retention was 93.28% when the rate increased from 0.1 C to 1 C, which is a very high capacity retention. Compared to the commercial PE films, rate-capability tests also carried out between the Li/SHPEM/LiCoO_2_ batteries and Li/PE (liquid electrolyte)/LiCoO_2_ batteries. As revealed in Figure 6c, a high capacity of 149 mAh·g^−1^ was achieved at the 0.1 C rate, which is as good as the Li/PE (liquid electrolyte)/LiCoO_2_ batteries, as well as 1 C rate.

In order to investigate the performance of the SHPEM when applied in a lithium ion battery, we created a cross scar by gently slicing the SHPEM samples using a fresh razor blade. After treatment at 80 °C under nitrogen for about 2 h, the healed SHPEM was assembled into a Li/SHPEM/LiCoO_2_ the battery for cycling stability tests. Figure 6d presents the cyclic stability of the Li/SHPEM/LiCoO_2_ batteries with the original and healed SHPE separator and commercial PE film and the schematic of the damaged and healed SHPE lithium battery membrane shown in Figure 6e. Compared with the battery Li/PE (liquid electrolyte)/LiCoO_2_, charged and discharged with a constant current of 140 mA·g^−1^ (1C rate) between 4.3 and 3.0 V, it can be seen from Figure 6d that the initial capacity of the Li/SHPEM/LiCoO_2_ battery is similar to that of the Li/PE (liquid electrolyte)/LiCoO_2_ battery and the cyclic stability of the Li/SHPEM/LiCoO_2_ battery is also as good as that of the Li/PE (liquid electrolyte)/LiCoO_2_ battery. Amazingly, the healed Li/SHPEM/LiCoO_2_ battery also exhibited excellent performance in the initial capacity test, indicating an ideal self-healing behavior. In addition, after 100 cycles, the Li/SHPEM/LiCoO_2_ battery maintains 96.1% of its initial discharge capacity, while the healed battery with SHP electrolyte membranes keeps 95.9% and the Li/PE (liquid electrolyte)/LiCoO_2_ battery keeps 96.9% of its initial discharge capacity. All of them have a high capacity retention, which demonstrates the excellent electrochemical stability of the self-healing batteries.

## 4. Conclusions

In summary, we have presented a new strategy for forming a SHP electrolyte membrane by constructing a thermally reversible polymer network with embedded electrolytes. Following the design strategy, we use a simple emulsion polymerization method to incorporate the advantages of high ionic conductivity, flexibility and self-healing ability, with cross-linking agents and embedding electrolytes, the prepared electrolyte membrane exhibited excellent electrochemical performance. By using the Diels-Alder cycloaddition reaction, we increased the tensile strength of the polymer and obtained the desired thermally reversibility properties, which could improve the safety performance of lithium-ion batteries. When the SHP electrolyte membrane was used as a separator in a lithium-ion battery with an LCO-based cathode, the SHP membrane achieved excellent rate capability and stable cycling for over 100 cycles, and the specific discharge capacity could be almost fully recovered after damage repair. Certainly the self-healing conditions could be further improved and optimized by changing the self-healing monomer. The successful preparation of this self-healing lithium-ion battery may provide a simple method to prepare battery separators with excellent electrochemical and safety performance, which will expand the lifetime of future energy storage devices and empower them with desirable economic and human safety attributes.

## Data Availability

Not applicable.

## Supplementary Materials

The following are available online at www.mdpi.com/article/10.3390/polym13234155/s1, Figure S1: TGA curve for cross-linked self-healing polymer from room temperature to 400 °C at a heating rate of 10 °C min^−1^, Figure S2: The Arrhenius plot for ionic conductivity as a function of temperature of the SHPEM (the range of the temperature from 90 °C to room temperature).
