# Supplementary material for: Self-Healing of a Covalently Cross-Linked Polymer Electrolyte Membrane by Diels-Alder Cycloaddition and Electrolyte Embedding for Lithium Ion Batteries
Source: Polymers (Basel). 2021 Nov 27;13(23):4155. doi: 10.3390/polym13234155 (PMC8659245; doi:10.3390/polym13234155)
Supplement: Supplementary file 1 [file polymers-13-04155-s001.zip › polymers-1422928-supplementary.pdf]

polymers

MDPI

Supporting Information

Self-Healing of a Covalently Cross-Linked Polymer Electrolyte Membrane by Diels-Alder Cycloaddition and Electrolyte Embedding for Lithium Ion Batteries

Lijuan Chen [1,†], Xisen Cai [1,†], Zhonghui Sun [1], Baohua Zhang [1], Yu Bao [1,*], Zhenbang Liu [1,*], Dongxue Han [1] and Li Niu [1,2]

[1] C/O Guangzhou Key Laboratory of Sensing Materials & Devices, Center for Advanced Analytical Science, School of Chemistry and Chemical Engineering, Guangzhou University, Guangzhou 510006, China; gdchenlj@gzhu.edu.cn (L.C.); 2111905009@e.gzhu.edu.cn (X.C.); cczhsun@gzhu.edu.cn (Z.S.); ccbhzhang@gzhu.edu.cn (B.Z.); dxhan@gzhu.edu.cn (D.H.); lniu@gzhu.edu.cn (L.N.)

[2] State Key Laboratory of Electroanalytical Chemistry, Changchun Institute of Applied Chemistry, Chinese Academy of Sciences, Changchun 130022, China

* Correspondence: baoyu@gzhu.edu.cn (Y.B.); cczbliu@gzhu.edu.cn (Z.L.)

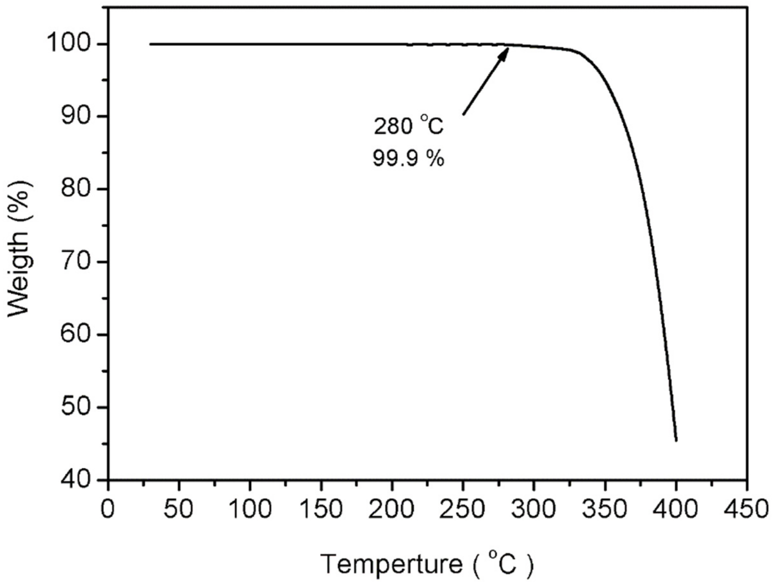

Figure S1. TGA curve for cross-linked self-healing polymer from room temperature to 400 °C at a heating rate of 10 °C min^{-1}.

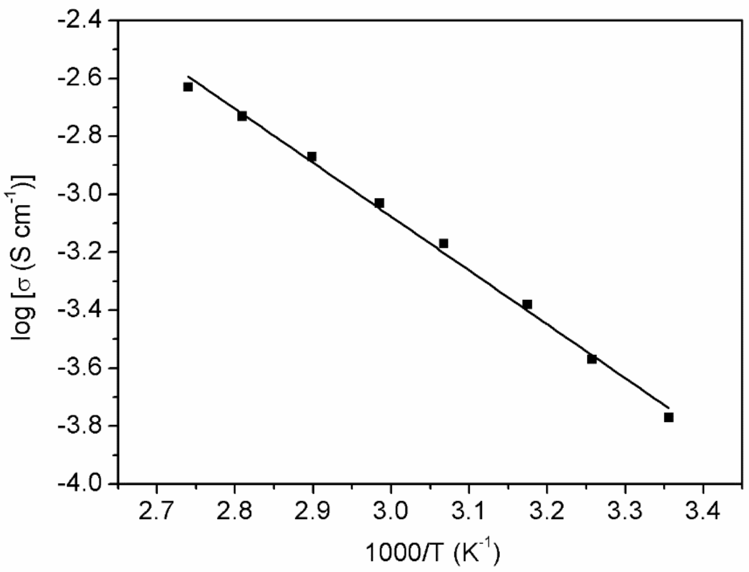

Figure S2. The Arrhenius plot for ionic conductivity as a function of temperature of the SHPEM (the range of the temperature from 90 °C to room temperature).
